# Trivialization of Aggression Against Women in India: An Exploration of Life Writings and Societal Perception

**DOI:** 10.3389/fpsyg.2022.923753

**Published:** 2022-07-07

**Authors:** S. Arya, Allen Joshua George

**Affiliations:** ^1^Department of English, School of Humanities and Languages, Central University of Karnataka, Gulbarga, India; ^2^Indian Institute of Management Ranchi, Ranchi, India

**Keywords:** life writing, verbal aggression, physical aggression, sexual aggression, normalization, trivialization, gendered aggression, domestic violence

## Abstract

**Purpose:**

Aggression, or an action that intend to harm, encompasses different forms with varying intensity, impact, and salient features. Globally and in Indian context specifically, aggression against women is often normalized if there is no physical aggression involved as the hurt caused tends to be invisible. The current study explored the perceived intensity of physical, verbal, and sexual aggression among south Indian adults.

**Method:**

Aggressive instances from the life writings of two south Indian women were chosen and were rated by five independent coders to check inter-coder reliability. The select narratives disclose instances of domestic aggression. Ten instances with highest ratings were chosen as the material for data collection. Adults (*N* = 145) from two southern states had reported the perceived intensity of aggression in each context. Textual analysis and ANOVA were the analytic techniques adopted.

**Results:**

The results indicate trivialization of verbal aggression compared to physical and sexual aggression. Further, the instances of verbal or more subtle aggression were perceived as even more trivial when the respondents got exposed to the instances of explicit physical and sexual aggressions first.

**Conclusions:**

The findings indicate trivialization of verbal and implicit forms of aggression, particularly when exposed along with physical and explicit forms of aggression. Consequences of different forms of aggression were not considered significant by the respondents. Suggestions for further studies, changes in policy-making, and law-enforcement were made based on the current results.

## Introduction

Aggression against women, often used by patriarchal societies to control women, is indeed a significant issue that still remains intense and frequent in the Indian society. Historically and cross-culturally, the overt use of physical and sexual violence has functioned as a key mechanism for perpetuating patriarchal control (Kreft, 2020; Santos et al., 2021). Further, studies also show that violence is not always perpetrated as a form of physical aggression but can also be psychological or verbal (Goessmann et al., 2021). Often while giving significance to physical aggression, as the hurt caused will be in most cases instantly visible, other forms of aggression like verbal and sexual aggression tend to be trivialized, marginalized or at times normalized.

Life Writing, defined by Leader (2015) as “a range of writings about lives or parts of lives, or which provide materials out of which lives or parts of lives are composed”, has been adopted by women writers as a medium to express resistance to the patriarchal ways of the society whereas tradition and culture continue to impose certain aggressive strategies against women to control them that further deem to be normal or acceptable. Domestic violence is frequently being perceived as ‘normal’ within different societies (Chester and Joscelyne, 2021). In the Indian context, mostly when women are being abused and their human rights violated, which cause both physical and psychological harm, the major reasons stem from societal traditions and culture (Dutt, 2018).

In Social Psychology, aggression is defined as “a behaviour that is intended to harm another person who is motivated to avoid that harm” (Allen and Anderson, 2017). Physical aggression implies causing physical harm that include kicking, punching, beating etc. whereas, using words to hurt others by calling names, yelling, screaming and so on is considered as verbal aggression (Bushman, 2019). Sexual aggression involves any forms of sexual behaviour toward a person who does not or cannot consent (Basile and Saltzman, 2002).

Cultural factors contribute to violence against women in India and the literature reports the torments women undergo for the sake of customs based on religious beliefs that may even result in death (Jaising, 1995). Interestingly, a systematic review reveals that the published quantitative studies for a decade report higher prevalence of physical abuse compared to psychological abuse and sexual abuse (Kalokhe et al., 2017). This further raises the question that whether physical aggression or violence is more prevalent or whether the society trivializes and normalizes forms of aggression other than physical aggression. In order to answer this question the current study has adopted textual analysis as well as a social survey to understand the nature of aggression perpetuated against women and the societal perception about the same.

### Purpose of the Study

The current study attempts to determine if verbal and sexual aggression tend to be trivialized in comparison with physical aggression in the context of domestic violence.

## Method

### Materials

For the current study, two text books—Jaishree Misra’s “Ancient Promises” (Misra, 2000) and Meena Kandasami’s “When I Hit You” (Kandasami, 2017)—where chosen. Initially, all major published life writings published between 2000 and 2020 by a contemporary South Indian female writer were considered for inclusion, out of which the books that did not elaborate on domesticity were excluded. The life writings with no English translation were also excluded. From the two text books, all instances where aggression against the protagonist was displayed were listed out. The list was given to five independent coders and asked to rate the suitability of its inclusion on a 10-point rating scale. They were also asked to label the instances as “Physical Aggression,” “Verbal Aggression,” and “Sexual Aggression.” Based on the highest scores, 10 instances (four Physical Aggression, four Verbal Aggression, and two Sexual Aggression, equally from both books) were chosen that obtained highest total rating from five coders. Additionally, two instances of non-aggression were also chosen, one from both texts. These two items were used as a “Lie Scale” and the scores of those participants who had scored one or above were excluded from the data for analyses, considering that they had either responded randomly or they did not comprehend the meaning of the items. The 12 items were presented to the respondents who were asked to rate the perceived intensity of each instance on a 11-point scale from “0” to “10”, “0” indicating “no aggression” and “10” representing “extreme aggression.”

An instance from “Ancient Promises”: *It didn’t take long for me to start hating myself for the many different things that gave the in-laws reason to slap their knees and laugh until tears ran down their cheeks. For my mother having omitted to teach me how to cook; for not being able to speak Malayalam elegantly; for forgetting constantly not to mind my Pleases and Thankyous; for having been brought up in Delhi; for having had an aunt who, in the nineteen- twenties, had an affair that everyone in Kerala(except me) had heard about. There was so much to be ashamed of.*

An instance from “When I Hit You”: *He kicks me in the stomach. ‘Prove it!’ he yells as I double over. ‘Prove it to me that you are my wife. Prove it to me that you are not thinking of another man. Or I will prove it for you.’ My hair is gathered up in a bunch in his hand now. He is lifting me by my hair alone. All the blood is rushing to my head, my thighs fight to feel the hard wood of the chair. I am in pain.*

### Participants

Adults from two south Indian states—Kerala and Tamil Nadu (*N* = 145; female = 108, male = 35, not disclosed = 2) had responded to 12 items that intended to assess their perception about different instances of aggression portrayed in the chosen text books. The participants were recruited through convenient sampling. Most of the participants (*N* = 113) belonged to the age 18-30, 30 participants were aged between 31 and 50, and the remaining two participants were over 51. A total of 83 individuals were unmarried, 60 of them married, and one person was divorced. Thirty-six participants hailed from rural regions, 47 from semi-urban areas, and 62 from urban areas. More sociodemographic details of the participants are given in tabular form as Supplementary Material.

### Procedures

Mixed method was used for the current study containing qualitative textual analysis and quantitative measures to assess the perception of participants about the intensity of aggressive instances described in the chosen texts. Considering the possibility of priming that might influence the participants’ responses, three forms of the assessment were prepared with different orders of presentation—(1) begins with instances from “Ancient Promises” (considerably mild verbalization of aggression) followed by the instances from “When I Hit You”; (2) begins with instances from “When I Hit You” (considerably intense and explicit expression of aggression); and (3) mixed. Based on the presentation order, participants were divided into Group 1, 2, and 3. Group 1 was administered with the mixed presentation of instances, Group 2 was given less intense instances first, and Group 3 was exposed with the intensely aggressive instances first.

### Remuneration

One randomly chosen participant each day were remunerated with INR (Indian Rupee) 50 if they responded to all the items and obtained zero “Lie Score.”

### Ethical Considerations

The participation was voluntary and all the participants were informed that the items contained sexual and aggressive instances. They were also informed that they were free to withdraw from the study at any point of time without giving a reason.

## Results

The results of the study are summarized in the following tables.

## Discussion

The current study has utilized life writings of select women that reflect the societal attitude about physical, verbal, and sexual aggression toward women, along with responses from a sample population from the same society who had exposed to the instances of violence depicted in these books. Both Misra’s and Kandasamy’s life writings reflect the aggression the authors had to endure after their marriage, and further narrates their efforts to come out of the abusive relationship. Reinharz posits voice as “having the ability, the means, and the right to express oneself, one’s mind, and one’s will. If an individual does not have these abilities, means, or rights, he or she is silent” (Reinharz, 1994). “To speak and to be heard is to have power over one’s life” (Ahrens, 2006). The select writers have overcome silence and reclaimed their voice opting to write that episode of their lives when they were mere victims of domestic abuse. Misra’s Ancient promises, a fictionalized biography, discusses the protagonist, Janaki’s, struggle to gain acceptance of her husband and in-laws. Janaki in the due course had to encounter mainly verbal aggression, and at times sexual and physical aggression. However, this very reason makes the writer trivialize the aggression she had to endure by comparing it with an acquaintance who is physically abused by her husband. In the narrative, she reiterates that the behaviours meted out at the in-laws are not serious enough to be complained about despite its adverse effects on her health and well-being. Kandasamy, on the other hand, has written more about physical and sexual aggression than verbal aggression. She attempts to resist the physical assaults and finds means to save herself rather than the marriage, unlike Misra. She was also more vocal about the aggression most probably because she had endured physical and sexual aggression that seemingly overpowered the verbal abuses.

The select life writings of renowned South Indian authors, Jaishree Misra and Meena Kandasamy reveals how aggression tends to be normalised in women’s everyday life, especially within the family. Misra’s “Ancient Promises” and Kandasamy’s “When I Hit You: The Portrait of the author as a Young Wife”, extensively narrate the episode of a difficult marriage which the authors had to confront, and their struggles to come out of it. Both the life writings expose different forms of aggression including but not limited to physical aggression, verbal aggression, relational aggression, and sexual aggression. This article, however, focuses on analysing the select instances of verbal, physical and sexual aggression from the text, as well as the readers’ responses to it. Attempts are made to understand whether the participants have identified the specific instance as aggression and to what extent.

The one-way ANOVA test results (Table 1) suggests that the participants’ perceived intensity of the three types of aggression was significantly different. Pots-Hoc test was carried out to see the specific differences among the types of aggression (Table 2) which indicates that all three categories of aggression differ significantly to each other. Verbal aggression was given the lowest score, sexual aggression obtained scores significantly higher than verbal aggression, and physical aggression had received the highest score.

**TABLE 1:** Comparison of the mean scores of physical aggression, verbal aggression, and sexual aggression.

		Sum of squares	df	Mean square	F	Sig.
Type of aggression	Between groups	2,858.611	2	1,429.306	22.256	0.000
	Within groups	27,744.000	432	64.222		
	Total	30,602.611	434			

**TABLE 2:** Post-Hoc test showing the difference among physical aggression, verbal aggression, and sexual aggression.

	Type of aggression	N	Subset for alpha = 0.05		
			1	2	3
Duncan^a^	Verbal aggression	145	25.6690		
	Sexual aggression	145		28.1379	
	Physical aggression	145			31.9034
	Sig.		1.000	1.000	1.000

*Means for groups in homogeneous subsets are displayed.*

*^a^Uses harmonic mean sample size = 145.000.*

The mean scores of three kinds of aggression indicate that individuals have perceived verbal aggression as less intense, and obviously less harmful, in comparison with physical aggression and sexual aggression. It is significant to note that the consequences of different forms of aggression were given little importance or the psychological consequences were given lesser importance than the physical and tangible consequences of aggression. Considering the culturally ingrained attitudes about aggression, particularly the belief that women who attempt to break the cultural norms deserve punishments in the form of aggression (Stephens and Eaton, 2020), the non-significant involvement of socio-demographic characteristics worth mentioning here, including gender. It indicates that both men and women support the suppression of women through various means and justify aggression against them, especially if it is not physical.

The select life writings, Jaishree Misra’s “Ancient Promises” and Meena Kandasamy’s “When I Hit You: The Portrait of the Writer as a Young Wife”, considered as semi biographical, brings to light several instances of domestic abuse. The books depict aggression of various types encountered by the protagonists. The text, thus, could be considered as a reflection of the aggression prevalent in the society and hence important documents to the study of aggression. As Coyne et al. (2011) argues, “though violence in media has been widely studied, there is a dearth in the study on aggression pervasive in books and its subsequent effect on the readers, despite its popularity.” Life writings not only reflect the societal attitudes and perceptions about aggression, but also likely to influence the readers by either promoting or questioning those attitudes.

Results represented by Table 3 reveals that based on the order of presentation, individuals had responded differently to aggressive instances. Specifically, as the result indicate, group 1, group 2, and group 3 differ significantly based in their scores on Verbal Aggression. Post-Hoc analysis was carried out to estimate the exact difference. Post-Hoc results (Table 4) indicate that Group II where the participants were exposed to the mildly described instances first, has perceived the instances as more aggressive compared to the other two groups.

**TABLE 3:** Comparison of the mean scores of group 1, group 2, and group 3 on physical aggression, verbal aggression, and sexual aggression.

		Sum of squares	Df	Mean square	F	Sig.
Physical aggression	Between groups	70.714	2	35.357	0.739	0.480
	Within groups	6,795.935	142	47.859		
	Total	6,866.648	144			
Verbal aggression	Between groups	635.627	2	317.813	6.743	0.002
	Within groups	6,692.484	142	47.130		
	Total	7,328.110	144			
Sexual aggression	Between groups	58.472	2	29.236	0.308	0.736
	Within groups	13,490.769	142	95.005		
	Total	13,549.241	144			
Total aggression	Between groups	1,622.126	2	811.063	2.034	0.135
	Within groups	56,609.708	142	398.660		
	Total	58,231.834	144			

**TABLE 4:** Post-Hoc test showing significant difference among group 1, group 2, and group 3 on basis of verbal aggression.

	Group	N	Subset for alpha = 0.05	
			1	2
Duncan^a^	Group 1	44	22.8182	
	Group 3	16	24.3750	
	Group 2	85		27.3882
	Sig.		0.374	0.087

*Means for groups in homogeneous subsets are displayed.*

*^a^Uses harmonic mean sample size = 30.930.*

The results signify the effect of priming observable through the significant difference among the responses of the individuals based on the order of presenting instances of different kinds of aggression. When the mildly or implicitly aggressive instances were presented first, participants perceived it as more intensely aggressive compared to the participants who had exposed to the explicitly aggressive instances first or a combination of both. This result emphasizes that comparing one’s personal experiences of verbal aggression with more explicit instances, or other forms of aggression, can make an individual believe that their situation is comparatively better than others. This trivialization of verbal aggression would promote tolerance of aggression that might encourage the perpetrators to become more intensely and frequently aggressive. It is important to note here that the same trend is not visible for physical aggression and sexual aggression where the aggressive behaviour is overt and the outcomes often more explicit. It need more elaborate researches to understand the exact role of priming and the involvement of any other variables in the trivialization of aggression.

In many different patriarchal societies and families, there is a general tendency to trivialize and normalize aggression against women within family, particularly among couples (Wood, 2001; Namy et al., 2017; Rodelli et al., 2022). Furthermore, the current study highlights the possibility of verbal aggression getting much more trivialized when there are comparative information about physical and sexual aggression. If reading about other forms of aggression, with or without personal experience, can depreciate verbal aggression, exposure to social media violence with vivid multisensory experiences can have tremendous undesirable consequences. Papp et al. (2022) report the association between media exposure and acceptance of patriarchal norm as well as sexualized aggression among women. Repeated exposure to media violence result in cognitive and emotional habituation and subsequent normalization of aggressive behaviours (Piotrowski and Fikkers, 2019). Further empirical studies are required to see if these normalization also include trivialization of verbal aggression. Nevertheless, trivialization of verbal aggression over physical aggression can result in serious psychological harm considering the potential impact of verbal aggression within families (Aloia, 2022). The current study advocates development of socio-psychological interventions and modifications in social policies to reduce trivialization of verbal aggression that pose a serious threat if unaddressed.

### Limitations and Further Directions

Current study is a preliminary exploration that may not be a true representation of the population, especially due to the sampling technique adopted. Also, the reasons and consequences of trivialization of aggression against women are also not identifiable from the current study. Again, the study does not clarify whether verbal and sexual aggression were considered by the participants as a form of domestic abuse. A qualitative or mixed research would answer these questions. Despite, it provides a general impression about the attitude of the society toward aggression against women that also corroborate with the life writings of the select authors. It is recommended to carry out further large-scale empirical researches to identify the reasons, extent, and impact of trivialized verbal aggression in familial contexts.

## Conclusion

Aggression against women is often trivialized in India, as it is evident from the current study results. Especially verbal aggression is considered as less intense with minor or negligible consequences. When tolerated, if verbal aggression will be considered as an approval that makes the perpetrator continue with similar or more intense forms of aggression is a question raised from the current findings. Similarly, whether the consequences of verbal, physical, and sexual forms of aggression can be equally harmful that destroy the healthy functioning of an individual also needs to be studied. In short, the current study results urge the need for early interventions to identify and control aggression of all kinds. Further, it demands attention from the academia, policy makers, women’s right activists, and the public to be aware of the consequences of all kinds of aggression including its various verbal forms. Also, the study signifies the imperative need of future studies to explore the exact impacts of verbal aggression on well-being, productivity, and health outcomes.

## Data Availability Statement

The raw data supporting the conclusions of this article will be made available by the authors, without undue reservation.

## Ethics Statement

Ethical review and approval was not required for the study on human participants in accordance with the local legislation and institutional requirements. The patients/participants provided their written informed consent to participate in this study.

## Author Contributions

SA did the conceptualization, prepared the materials for data collection, carried out the qualitative textual analysis, and contributed in the data collection and writing the manuscript. AG prepared the research design and done the quantitiave analysis and contributed in data collection and the writing of the manuscript. Both authors contributed to the article and approved the submitted version.

## Conflict of Interest

The authors declare that the research was conducted in the absence of any commercial or financial relationships that could be construed as a potential conflict of interest.

## Publisher’s Note

All claims expressed in this article are solely those of the authors and do not necessarily represent those of their affiliated organizations, or those of the publisher, the editors and the reviewers. Any product that may be evaluated in this article, or claim that may be made by its manufacturer, is not guaranteed or endorsed by the publisher.
