# Supplementary material for: Trivialization of Aggression Against Women in India: An Exploration of Life Writings and Societal Perception
Source: Front Psychol. 2022 Jul 7;13:923753. doi: 10.3389/fpsyg.2022.923753 (PMC9301204; doi:10.3389/fpsyg.2022.923753)
Supplement: Supplementary file 1 [file Data_Sheet_1.docx]

Table 1: Socio-demographic characteristics of the participants

| **Socio-demographics** | | **(N=145) %** |
| --- | --- | --- |
| Gender |  |  |
|  | Female | 108 (74.5) |
|  | Male | 35 (24.1) |
|  | Prefer not to say | 2 (1.4) |
| Education |  |  |
|  | Up to 12^th^ Grade | 12 (8.3) |
|  | Graduation | 34 (23.4) |
|  | Professional Degree | 12 (8.3) |
|  | Post Graduation | 77 (53.1) |
|  | PhD/ Post-Doc | 10 (6.9) |
| Marital status |  |  |
|  | Unmarried | 83 (57.2) |
|  | Married | 60 (41.4) |
|  | Divorced/ Separated | 1 (1.4) |
| Place of residence |  |  |
|  | Rural | 36 (24.8) |
|  | Semi-urban | 47 (32.4) |
|  | Urban | 62 (42.8) |
| Age |  |  |
|  | 18-30 | 113 (77.9) |
|  | 31-50 | 30 (20.7) |
|  | 51 and above | 2 (1.4) |
| Occupation |  |  |
|  | Student/ Research Scholar | 72 (49.7) |
|  | Unemployed/ Homemaker | 19 (13.1) |
|  | Self-employed | 12 (8.3) |
|  | Private sector | 32 (22.1) |
|  | Government sector | 10 (6.9) |

**Instances from Meena Kandasamy’s ‘When I Hit You’.**

1. One of the select instances reveals severe physical aggression perpetuated by the husband which the wife had to endure. The writer notes that citing silly reasons and accusations, he would initiate arguments which would escalate into physical aggression. She describes herself at a vulnerable position receiving the ‘torrent of blows’. It is significant that the instance further shows how the physical aggression instilled absolute fear in the protagonist.

2. In the second instance, Kandasamy writes how the wife was humiliated and further her morality was questioned just for having received an opportunity from a reputed journal to write an essay on sexuality. Belittling her, the husband claims that her expertise of having maintained sexual relationship with other men belonging to different age groups have contributed in fetching her write the article on the specified topic. The protagonist asserts that ‘she would be unable to share this accusation with any one and the readers would remain unaware about the shaming she had endured while attempting to write the article’. In the current context, the protagonist endures verbal aggression. Her husband devalues her skills and further attempts to portray her as a ‘whore’.

3. In the third select instance, Kandasamy divulges the protagonist having encountered sexual aggression. She narrates how the protagonist had frequently endured marital rape and the adverse effects it had on her. It is observed in the narrative that being constantly raped eventually “extended the threshold of pain, shame and brutality”.

4. The fourth select instance in the narrative notes how the husband undermined the wife’s prowess as a writer by denouncing her as insane and further labelling her as immoral. Her attempts to retort fails in front of his false accusations. Here, verbal aggression is employed to dishonor her and crush her confidence.

5. The last instance from the select narrative shows how the protagonist is severely battered by her husband. He kicks and further lifts her by the hair.

**Instances from Jaishree Misra’s ‘Ancient Promises’.**

1.Being informed about the daughter’s growing romantic relationship with a boy, her mother tries to dissuade her. Suspicious of her true intend, here the mother questions Janaki’s morality. This, an instance of verbal aggression, is employed to coerce Janaki to get into an arranged marriage discarding her ideas to continue studies or sustain the affair.

2.The second given instance narrates the consummation of marriage, where Janaki, being Suresh’s wife points it out ‘as one of those things that had to be done’ after which she gives a glimpse to the readers about her complex emotion. Poignantly, Janaki recollects “I tried to quell the feeling of revulsion that rose in my chest”. It is visible that Janaki does not have any agency over her body. Her understanding that a wife must submit to her husband, even sexually, further prompts her to remain passive and submissive to her husband’s sexual advancements despite her aversion which in turn make her feel disgusted. This instance reveals how the cultural expectations and further the privilege conferred on the husband ignores woman’s consent, coercing her to yield to him as per his desire.

3.In the third instance, Misra portrays Janaki struggles to feel included and accepted by her husband’s family. However, they humiliated her persistently citing numerous reasons including her inability to converse eloquently in the native language, for her aunt’s controversial affair, for her mother having forgotten to teach her to cook and so on. This in turn made Janaki feel ‘ashamed’ and isolated. Here verbal aggression, targeted against the protagonist, adversely affects her and makes her feel as an intruder in her husband’s home.

4.Suresh develops plans to depict his wife, Janaki, as mentally unstable and further admit her to a mental asylum. He considers it better to have a wife who is insane rather than one who seeks divorce, questioning his masculinity and tarnishing the reputation of his family. The narrative further shows Janaki, forcefully sedated and admitted to a hospital where she is pronounced as a maniac and suffering from delusions. The episode also reveals her inability to talk or move because of the powerful medication. In this instance the husband turns physically aggressive and forcefully admits her to the hospital.

5.In the fifth select instance from the narrative, Janaki is at the receiving end of her father’s anger for having found her growing affair with Arjun. Janaki was caned and then coerced to refrain from engaging in the romantic relationship. Here the father resorts to corporal punishment to instill fear in his daughter, control and further make her obey him.
